# Neuronal and Astrocytic Regulations in Schizophrenia: A Computational Modelling Study

**DOI:** 10.3389/fncel.2021.718459

**Published:** 2021-08-26

**Authors:** Lea Fritschi, Johanna Hedlund Lindmar, Florian Scheidl, Kerstin Lenk

**Affiliations:** ^1^Department of Mathematics, ETH Zurich, Zurich, Switzerland; ^2^Institute of Neuroinformatics, University of Zurich, ETH Zurich, Zurich, Switzerland; ^3^Department of Computer Science, ETH Zurich, Zurich, Switzerland; ^4^Computational Biophysics and Imaging Group (CBIG), Faculty of Medicine and Health Technology, BioMediTech, Tampere University, Tampere, Finland; ^5^Institute of Neural Engineering, Graz University of Technology, Graz, Austria

**Keywords:** computational psychiatry, computational model, neuron-astrocyte network, glutamate, schizophrenia, linear mixed effects models

## Abstract

According to the tripartite synapse model, astrocytes have a modulatory effect on neuronal signal transmission. More recently, astrocyte malfunction has been associated with psychiatric diseases such as schizophrenia. Several hypotheses have been proposed on the pathological mechanisms of astrocytes in schizophrenia. For example, post-mortem examinations have revealed a reduced astrocytic density in patients with schizophrenia. Another hypothesis suggests that disease symptoms are linked to an abnormality of glutamate transmission, which is also regulated by astrocytes (glutamate hypothesis of schizophrenia). Electrophysiological findings indicate a dispute over whether the disorder causes an increase or a decrease in neuronal and astrocytic activity. Moreover, there is no consensus as to which molecular pathways and network mechanisms are altered in schizophrenia. Computational models can aid the process in finding the underlying pathological malfunctions. The effect of astrocytes on the activity of neuron-astrocyte networks has been analysed with computational models. These can reproduce experimentally observed phenomena, such as astrocytic modulation of spike and burst signalling in neuron-astrocyte networks. Using an established computational neuron-astrocyte network model, we simulate experimental data of healthy and pathological networks by using different neuronal and astrocytic parameter configurations. In our simulations, the reduction of neuronal or astrocytic cell densities yields decreased glutamate levels and a statistically significant reduction in the network activity. Amplifications of the astrocytic ATP release toward postsynaptic terminals also reduced the network activity and resulted in temporarily increased glutamate levels. In contrast, reducing either the glutamate release or re-uptake in astrocytes resulted in higher network activities. Similarly, an increase in synaptic weights of excitatory or inhibitory neurons raises the excitability of individual cells and elevates the activation level of the network. To conclude, our simulations suggest that the impairment of both neurons and astrocytes disturbs the neuronal network activity in schizophrenia.

## 1. Introduction

Astrocytes contribute to the complex cognitive function in humans (Santello et al., 2019), whereby they have structural, homeostatic, and metabolic roles. Astrocytes are connected to each other via gap junctions and form non-overlapping domains in complex networks. This glial cell type composes, together with a pre- and a postsynaptic neuron, the so-called tripartite synapse (Araque et al., 1999). They take up glutamate rapidly from the synaptic cleft to ensure a short glutamate exposition to the postsynaptic neuron for precise synaptic transmission. Subsequently, astrocytes respond to neuronal activity with intracellular calcium (Ca^2+^) elevations and release ions and transmitter molecules in return.

Seemingly neurons and astrocytes play a role in psychiatric diseases such as schizophrenia (SCZ) (Uhlhaas and Singer, 2010; Takahashi and Sakurai, 2013; Moraga-Amaro et al., 2014; Mei et al., 2018). SCZ is associated with positive (such as delusions and hallucinations), negative (such as social withdrawal and lack of motivation), and cognitive symptoms (difficulties in memory and attention).

Due to the success of first generation antipsychotics, the *dopamine hypothesis of SCZ* was formed. The underlying assumption of this hypothesis is that excessive transmission of subcortical dopamine causes the positive symptoms of SCZ (Grnder and Cumming, 2016). While research has found significant support of this hypothesis, it is not pathognomonic and does not sufficiently account for the negative symptoms of SCZ.

Post-mortem examinations, human induced pluripotent stem cell experiments, and animal studies have revealed a reduced astrocytic and neuronal synaptic density in SCZ patients (Cotter et al., 2001; Brennand et al., 2011; Dietz et al., 2020; Naujock et al., 2020). Cell death is associated with excitotoxicity due to excessive glutamate amounts and glutamate receptor activation (Drouin-Ouellet et al., 2011). However, the mechanisms and pathways of this developmental brain disorder are not well understood yet. Different studies reach conflicting conclusions. Some papers report an upregulation in glial fibrillary acidic protein (GFAP) in SCZ astrocytes (review by Moraga-Amaro et al., 2014), whereas in other studies, a reduced GFAP expression has been described (review by Takahashi and Sakurai, 2013).

Koskuvi et al. (2020) found that human stem cell-derived astrocytes from SCZ patients show highly sex-specific alterations in gene expression. The Gene Ontology pathways related to neuronal wiring and inflammation are altered in SCZ astrocytes. Healthy neurons co-cultured with SCZ astrocytes of either sex exhibited a significantly increased response to glutamate. Transplanted hiPSC-astrocyte progenitors from SCZ patients developed into matured astrocytes in a mouse model and led to subtle behavioural changes. Furthermore, they induced demyelination, synaptic dysfunction and affected the inflammation pathways (Koskuvi et al., 2020). Several studies have shown that the expression of the metabotropic glutamate receptor (mGluR) type 5 is increased in astrocytes in the prefrontal cortex (Ohnuma et al., 1998; Kosten et al., 2016).

For neurons, the *glutamate hypothesis* of SCZ has been established (de Bartolomeis et al., 2005; Moghaddam and Javitt, 2012; Takahashi and Sakurai, 2013; Cohen et al., 2015; Mei et al., 2018): a hypofunction of NMDA receptors (NMDARs) leads to excessive glutamate release, mainly prevalent in the hippocampus and prefrontal cortex. However, the underlying mechanisms are not yet clarified. A decreased level of D-serine, which is mainly released by astrocytes, affects the NMDAR function in neurons. Both glutamate and either D-serine or glycine are co-agonists to the NMDAR binding sites. The astrocyte-derived NDMAR antagonist kynurenic acid (KYNA) shows increased levels in SCZ patients (Takahashi and Sakurai, 2013; Cohen et al., 2015). Interneurons might react to changes in NMDAR function with inhibition, leading to disinhibition and a hyperexcitation of glutamate levels in SCZ (Moghaddam and Javitt, 2012; Mei et al., 2018). Alternatively, Schobel et al. (2013) suggested that excess synaptic glutamate release results in a reduction of the interneuronal function and hippocampal disinhibition. The abnormality of glutamate transmission is also related to astrocytes since they regulate the glutamate uptake and release from and to the extracellular space (Danbolt, 2001; Kondziella et al., 2007). While the glutamate hypothesis is more recent and less well studied than the dopamine hypothesis, it has the advantage of being able to account for a wider range of SCZ symptoms (Hu et al., 2015; Mei et al., 2018).

A third hypothesis is that, instead, the excessive glutamate release leads to the NMDAR hypofunction. Higher glutamate levels may result in a rise of astrocytic Ca^2+^ levels. Subsequently, astrocytes may release higher concentrations of the gliotransmitter adenosine triphosphate (ATP). Lalo et al. (2016) suggested that the activation of postsynaptic purinergic P2X receptors (P2XRs) by ATP downregulates NMDARs.

These findings indicate that different neuronal and astrocytic malfunctions could lead to the same disease, despite different underlying mechanisms. This poses a major challenge to effective treatments for psychiatric diseases (Herman et al., 2012; Stephan et al., 2016). Computational models can complement or supersede experimental studies (Manninen et al., 2018; Oschmann et al., 2018), where the latter are not feasible or too expensive. They can guide experimental researchers to the relevant pathways that are altered in disease and thus predict pharmaceutical responses (Moran et al., 2016). Siekmeier and Hoffman (2002) proposed a computational model in which they reduced the number of synapses in a neuronal network. This synaptic pruning leads to semantic priming and difficulties in accessing memories in SCZ patients. Mahdavi et al. (2014) developed a tripartite synapse model focusing on the dysregulation of D-serine released by the astrocyte. To our best knowledge, no biophysical neuron-astrocyte network models have been published that explore the role of astrocytes in SCZ.

In this study, we investigate how changes in mechanisms and topology in neuron-astrocyte networks alter the functional state, i.e., spike and burst rate of neurons as well as glutamate levels in astrocytes, in health and SCZ. For this, we use the previously published computational model called INEXA (Lenk et al., 2020) to test the following four SCZ-related hypotheses: we alter (1) the number of neurons or astrocytes in the network, (2) the effect of astrocytic ATP on the postsynaptic activity, (3) the release of glutamate from the presynapse and the uptake of glutamate by the astrocyte, and (4) the excitatory and/or inhibitory synaptic strength.

## 2. Materials and Methods

**Figure 1:**
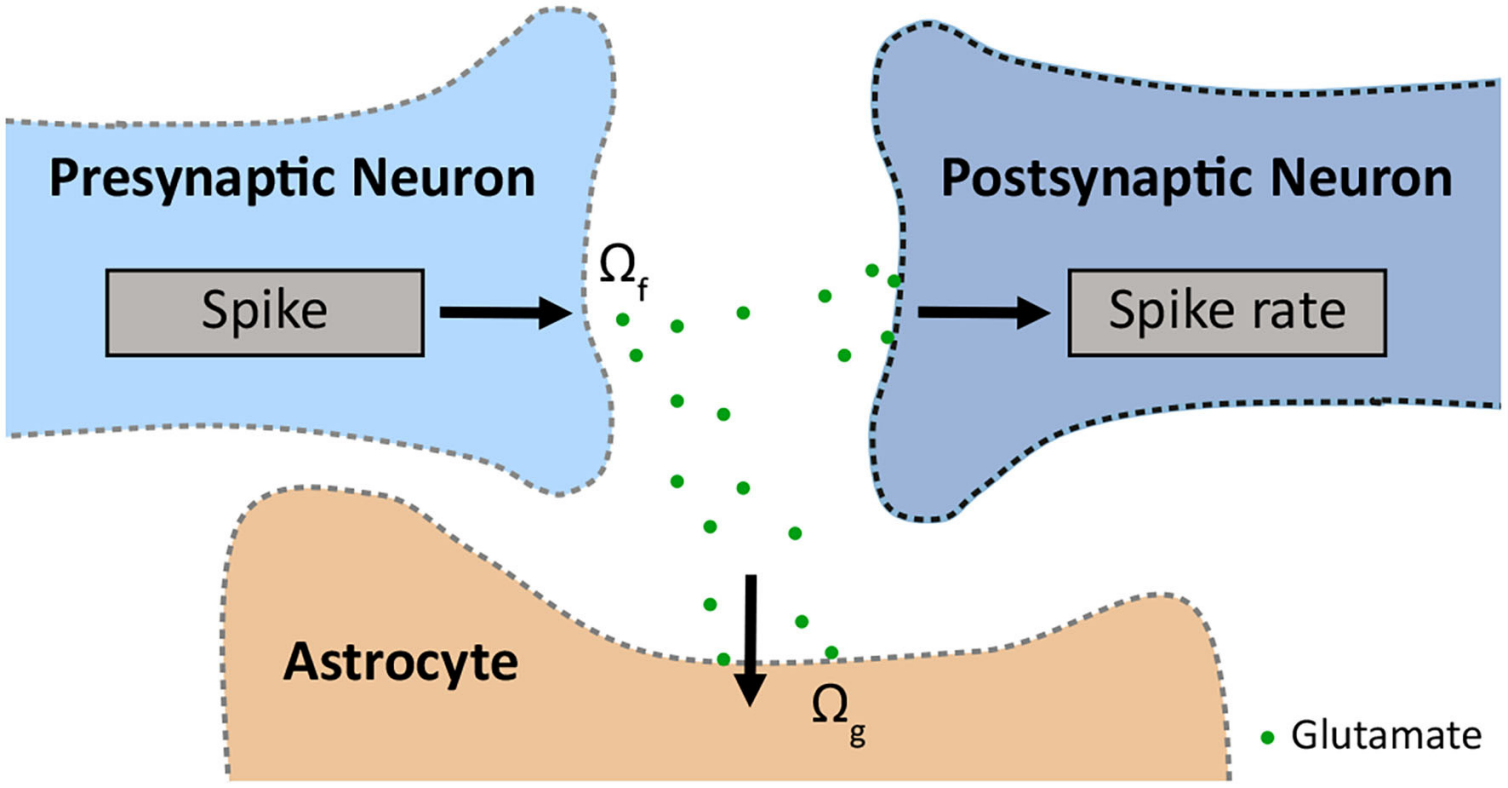
Schematic of the tripartite synapse, which consists of a pre- and a postsynapse and an astrocyte, modelled with INEXA (Lenk et al., 2020). The presynapse releases glutamate with release rate Ω_*f*_. The glutamate is taken up by the astrocyte with uptake rate Ω_*g*_.

To study the three aspects mentioned above in SCZ, we used the published model INEXA (Lenk et al., 2020) with the main components as shown in Figure 1. In section 2.1, we describe the governing equations of the INEXA model. In section 2.2, we explain how the model is used to simulate neuron-astrocyte networks in healthy and SCZ states. In sections 2.3, 2.4, the statistical analysis of the resulting neuronal and astrocytic activity is described.

### 2.1. Governing Equations of the INEXA Model

The phenomenological model INEXA (Lenk et al., 2020) includes both excitatory and inhibitory neurons and was initially developed to study the astrocytes' effect on neuronal firing rates in the cortex. The governing equation for simulating neurons is the one for the firing rate λ_*i*_ of a postsynaptic neuron *i* for each time step *t*_*k*_ of 5 *ms*:

(1) $$\begin{array}{l} \lambda_{i}\left(t_{k}\right)=\max\left(0,c_{i}+\underset{j}{\sum }y_{ij}\cdot s_{j}\left(t_{k-1}\right)-\underset{j}{\sum }y_{\text{Astro}}\cdot A_{ija}\left(t_{k-1}\right)\right), \end{array}$$

where *c*_*i*_ is the stochastic noise of neuron *i*. The term *y*_*ij*_ denotes the synaptic weight between the presynaptic neuron *j* and a postsynaptic neuron *i*, which can vary at each time step. The synapse can be either excitatory (*y*_*ij*_ ∈ [0, 1], representing glutamatergic neurons) or inhibitory (*y*_*ij*_ ∈ [−1, 0], representing GABAergic interneurons). The parameter *s*_*j*_ is a binary term indicating whether neuron *j* transmitted a spike in the previous time step *t*_*k*−1_. The second term in the equation denotes the depressing effect caused by astrocytes. The variable *A*_*ija*_ is binary, indicating whether a synapse *ij* is enclosed by astrocyte a and if astrocyte a was active at the previous time step. If this is the case, the astrocyte applies a depressing effect, *y*_Astro_, on the synapse. Otherwise, *A*_*ija*_ is zero, removing the astrocytic effect on neuronal activity.

Due to limited experimental data on the interaction between GABAergic neurons and astrocytes, the INEXA model currently only connects astrocytes with glutamatergic synapses. These excitatory synapses release glutamate into the synaptic cleft with rate Ω_*f*_. The released glutamate binds to astrocytic mGluRs with rate Ω_*g*_ causing the inositol 1,4,5-trisphosphate (IP_3_)-mediated release of Ca^2+^ from the endoplasmic reticulum (ER) into the astrocytic cytoplasm (Figure 1).

The governing equation for astrocyte dynamics describes the intracellular calcium dynamics ${\left[Ca^{2+}\right]}_{ija}$ in an astrocyte a that encloses the synapse *ij*:

(2) $$\begin{array}{l} {\left[Ca^{2+}\right]}_{ija}\left(t_{k}\right)={\left[Ca^{2+}\right]}_{ija}\left(t_{k-1}\right)+\Omega_{acc}\cdot ({\left[IP_{3}\right]}_{ija}\left(t_{k}\right)\\ \;-{\left[Ca^{2+}\right]}_{ija}\left(t_{k-1}\right)). \end{array}$$

The calcium concentration is composed of the calcium concentration left from the last time step ($Ca_{ija}^{2+}\left(t_{k-1}\right)$), the IP_3_-mediated Ca^2+^-induced Ca^2+^-release from the ER stores, and the Ca^2+^ uptake back to the ER by the SERCA (sarco/endoplasmic reticulum Ca^2+^-ATPase) pumps. To reflect the slow dynamics of the calcium release (which can take up to multiple seconds), we multiplied the ER term by the time scale Ω_*acc*_. A more detailed description of the INEXA model can be found in the original article (Lenk et al., 2020).

### 2.2. Simulated Experimental Setups

The topology and connectivity of the neuron-astrocyte network models were generated as described in Lenk et al. (2020) (see parameters in Table 1). Hence, 107 astrocytes and 250 neurons were placed uniformly at random on a planar area of 750 × 750 × 10 μ*m*^3^, resembling the electrode field of an *in vitro* multielectrode array (MEA). Neurons and astrocytes within a distance of 30 μ*m* were reallocated to account for the cell sizes. Astrocyte-astrocyte connections were formed whenever the distance was smaller than 100 μ*m*. Neuron-neuron and astrocyte-neuron connections were created according to a truncated Gaussian distribution based on their distance.

**Table 1:** Used parameters for simulating healthy and SCZ neuron-astrocyte networks.

**Parameter**	**Value**	**Unit**	**Definition**
$Y_{\max}^{+}$	**0.7**;0.8;0.9	-	Upper boundary for excitatory synaptic weights
$Y_{\max}^{-}$	0.1;0.5;0.6;**0.7**	-	Upper boundary for inhibitory synaptic weights
Ω_*f*_	**0.0020**;0.0010;0.0005	*s* ^−1^	Rate of synaptic facilitation
Ω_*g*_	**0.0770**;0.0385;0.0193	*s* ^−1^	Recovery rate of gliotransmitter receptors
*y*_Astro_	**0.01**;0.025;0.05;0.075;0.1	-	Downregulation of NMDARs by P2XRs
Astrocytes	81;**107**	-	Number of astrocytes in the network
Neurons	187;**250**	-	Number of neurons in the network

*Bold printed numbers refer to the values used in Lenk et al. (2020)*.

To study the effects of various spatial network topologies and network connectivities, we generated three spatially different network topologies A, B, and C. For each network A, B, C, we computed three independent connectivity configurations (CC 1, 2, 3) according to the rules described above, resulting in a total of nine different networks. We considered those networks to represent the healthy, non-pathological baseline. Different topological properties of these initial networks are described in Table 2. The stochastic noise *c*_*i*_ was set to 0.02 in all simulations.

**Table 2:** Different connectivity properties of the nine initial networks used in this paper.

**Network &**	**Network**	**Average number of**	**Excitatory synapses**
**CC**	**connectivity [%]**	**gap junctions**	**w.o. astrocyte [%]**
A 1	27.32	4.88	8.75
A 2	27.42	4.88	8.72
A 3	27.43	4.88	8.55
B 1	27.53	4.50	1.75
B 2	27.57	4.50	1.77
B 3	27.59	4.50	1.90
C 1	28.93	4.79	5.65
C 2	29.16	4.79	5.46
C 3	28.95	4.79	3.36

*In the first column, A, B, C refer to three networks with the same topology features. The numbers indicate the varying neuron-neuron and astrocyte-neuron network connectivity configurations (CC 1, 2, 3). The network connectivity (second column) denotes the percentage of neuronal connections formed. The average number of gap junctions (third column) describes how many connections each astrocyte forms with other astrocytes on average. Since the astrocyte-astrocyte network connectivity is not probabilistic, this value is constant for each network with the same underlying spatial topology. Excitatory synapses without astrocyte (fourth column) expresses the percentage of excitatory neuronal synapses left without an astrocyte*.

To simulate healthy, non-pathological neuron-astrocyte network activity, we tuned the parameters of the INEXA model so that they resemble *in vitro* MEA recordings. Thereby, we used a reference spike rate of 30–200 spikes per minute as measured from mouse neurons (Gramowski et al., 2006; Jenkinson et al., 2017) and human-derived neurons (Tukker et al., 2018; Kizner et al., 2019).

Based on hypotheses and experimental evidence about SCZ described in the Introduction, we modified four different sets of INEXA parameters and examined their effect on neuronal and astrocytic activity. More specifically, we simulated the following four pathological changes in the neuron-astrocyte interaction:

1. We stochastically removed 25% of astrocytes or 25% of neurons from premade network configurations to account for the reduced astrocytic and neuronal synaptic density in SCZ patients.
2. We amplified the depressing signal *y*_Astro_ of astrocytes to study the effect of astrocytic ATP on the postsynaptic activity.
3. We scaled the two parameters Ω_*f*_ and Ω_*g*_ by the factors 0.5 and 0.25, which control glutamate release from presynapses and glutamate uptake by astrocytes, respectively (Figure 1). Simulations were performed for each combination of the distorted and unmodified parameter values Ω_*f*_ and Ω_*g*_.
4. We increased the maximal synaptic weight $Y_{max}^{+}$ and $Y_{max}^{-}$ for excitatory and inhibitory neurons, which both result in a higher excitability of the cells.

The latter two manipulations allowed us to simulate the excessive release of glutamate in excitatory neurons and astrocytes as well as the dysfunctional interneurons assumed in the *glutamate hypothesis of SCZ*. A summary of the modified parameters can be found in Table 1. All other model parameters not listed in this table were used as described in the original paper by Lenk et al. (2020). Each simulation consisted of *T* = 5*min* simulated time and was repeated 10 times to account for the statistical variability of our results.

### 2.3. Quantifying Neuronal and Astrocytic Activity Features

To analyze the neuronal activity, we calculated spike and burst describing features. Bursts are cascades of spikes (i.e., action potentials). The features were determined using a modified version of the cumulative moving average (CMA) algorithm (Kapucu et al., 2012; Välkki et al., 2017). Therewith, we calculated the *mean spike rate* in spikes per minute and the *mean burst rate* in bursts per minute across all neurons, respectively. Additionally, we quantified the astrocytic activity by calculating how often an astrocyte changes its state to ‘active’ during the simulated time *T* and denoted the count as *mean number of activations per astrocyte*. We will refer to these three features as response variables below. The results were depicted as box plots.

### 2.4. Testing the Effects of Different Neuronal and Astrocytic Dysfunctions

The objective of this section is twofold: we quantify the effects of the four simulated pathological changes in SCZ (see section 2.2) on the neuronal and astrocytic response variables and estimate how much the effects vary for different network configurations. In the previously described simulated experimental setups, there were two sources of stochasticity. One came from the stochastic nature of the INEXA model, e.g., when creating the excitatory and inhibitory synaptic weights and in the update equations. The other was due to the fact that neuron-neuron and astrocyte-neuron connections were formed stochastically in our setup. To estimate the average random effect from both sources of stochasticity, we performed all simulations on the nine initial networks, as described in section 2.2. We analysed the data with linear mixed-effects (LME) models using the lme4-package (Bates et al., 2015) in R, version 3.6.3 (2020-02-29). In order to satisfy the LME model requirements, it was sometimes necessary to transform the response variable *Z* by some function φ:ℝ → ℝ. In the following subsections, we decomposed the variance of φ(*Z*) into intra-network terms Σ, which capture the stochasticity from different network connectivity configurations, and the residual error variance σ^2^. We note that these are the variance terms for the transformed response φ(*Z*). If one is interested in prediction intervals for *Z*, these must first be computed for φ(*Z*) and then back-transformed.

#### 2.4.1. Hypothesis 1: Astrocyte or Neuron Removal

In the first set of simulated experiments, we quantified the average effect of stochastically removing 25% of the astrocytes or neurons, respectively. In order to estimate the random effect of different network configurations, we made three identical copies of each of the nine networks described in section 2.2 and Table 2. Then, we either randomly removed 25% of astrocytes or 25% of neurons including their connections to other cells, which resulted in 27 cell-reduced networks per cell type.

For all of these networks, we ran 10 simulations and fit the resulting data using an LME model with a fixed effect for cell removal and a random effect for different network configurations. Using contrasts to distinguish the full network from reduced networks (Oehlert, 2010) (see Supplementary Material), we then determined the average reduction δ_rem_ of the response variables resulting from cell removal in percent. Moreover, we decomposed the variance of the transformed response φ(*Z*) into an inter-network term $\sigma_{\text{rem}}^{2}$ and the residual error variance σ^2^. For each response variable, we report the restricted maximum likelihood (REML) estimates, 95% confidence intervals (CIs) of δ_rem_ as well as the *p*-value for the null-hypothesis that the mean response of reduced and default networks A, B and C coincides. We also present the REML estimates and the 95% CIs for the standard deviations $\sigma_{\text{rem}}/\hat{\mu }$ and $\sigma /\hat{\mu }$, which have been normalised by the mean $\hat{\mu }$ of the transformed response φ(*Z*), when no cells were removed. A more detailed description of the reported quantities mentioned in this section and the LME model can be found in the Supplementary Material.

#### 2.4.2. Hypothesis 2: Effect of Astrocytic ATP

In this part, we altered the impact of astrocytic ATP on the postsynaptic P2XR and NMDAR activity by modifying the parameter *y*_Astro_. Therefore, we first performed 10 simulations with *y*_Astro_ ∈ {0.01, 0.025, 0.05, 0.075, 0.1} for each of the nine networks described in section 2.2. To analyze our simulated data, we used the LME model

(3) $$\begin{array}{l} \log\left(Z_{ij}\right)=\left(\mu +u_{i,\mu }\right)+\left(\beta_{a}+u_{i,a}\right)\cdot y_{\text{Astro}}+\beta_{a^{2}}\cdot y_{\text{Astro}}^{2}+\varepsilon_{ij}, \end{array}$$

where the index *i* enumerates the nine network configurations and *j* ∈ {1, …, 10} indices the 10 independent repetitions. The variable μ is the average value of log(*Z*) if *y*_Astro_ was equal to zero. The coefficients $\beta_{a},\beta_{a^{2}}$ correspond to the average increase of log(*Z*) in dependence of *y*_Astro_ and $y_{\text{Astro}}^{2}$, respectively. Random effects are captured in the vectors *u*_*i*_: = (*u*_*i*, μ_, *u*_*i,a*_) for each *i*, which are independent with multivariate Gaussian distribution $u_{i}\sim N\left(0,\Sigma \right)$ with mean **0** and diagonal covariance matrix $\Sigma =\mathrm{diag}\left(\sigma_{\mu }^{2},\sigma_{a}^{2}\right)\in {\mathbb{R}}^{2\times 2}$. This means that we assumed the individual components to be independent. The stochasticity of the INEXA model and the residual error is captured in ε_*ij*_, which we assumed to be normally distributed with mean 0, equal variance σ^2^ and independent of *u*_*i*_ for all indices *i*. We reported REML estimates and CIs for $\mu ,\beta_{a},\beta_{a^{2}},\Sigma$ and σ^2^.

#### 2.4.3. Hypothesis 3: Glutamate Dynamics Parameters

Following the *glutamate hypothesis of SCZ*, we tested the effect of changing the parameters regulating the glutamate dynamics in neurons and astrocytes. The parameters *w*_*f*_ and *w*_*g*_ for the glutamate dynamics are defined as follows: running the LME model with parameter values *w*_*f*_ and *w*_*g*_ corresponds to the scaling of the presynaptic glutamate release rate by a factor *w*_*f*_ (equivalent to a multiplication of Ω_*f*_ by *w*_*f*_) and scaling the astrocyte glutamate uptake rate by a factor *w*_*g*_ (equivalent to multiplying Ω_*g*_ by *w*_*g*_) compared to the default rates of the INEXA model. For every response variable *Z* = *Z*(*w*_*f*_, *w*_*f*_), we used the LME model

(4) $$\begin{array}{l} Z_{ij}=\left(\mu +u_{i\mu }\right)+\left(\beta_{f}+u_{i,f}\right)w_{f}+\left(\beta_{g}+u_{i,g}\right)w_{g}+\beta_{f^{2}}w_{f}^{2}\\ \;+\beta_{g^{2}}w_{g}^{2}+\left(\beta_{fg}+u_{i,fg}\right)w_{f}w_{g}+\varepsilon_{ij}, \end{array}$$

where the index *i* enumerates the nine network configurations described in section 2.2 and *j* ∈ {1, …, 10} denotes 10 independent repetitions. The variable μ stands for the average value of the response *Z* if *w*_*f*_ and *w*_*g*_ were equal to zero. The coefficients $\beta_{f},\beta_{g},\beta_{f^{2}},\beta_{g^{2}},\beta_{fg}\in \mathbb{R}$ correspond to the average increase of *Z* in dependence of the parameters they are multiplied by. Random effects were captured in vectors *u*_*i*_: = (*u*_*i*, μ_, *u*_*i,f*_, *u*_*i,g*_, *u*_*i,fg*_), which are independent with multivariate Gaussian distribution $u_{i}\sim N\left(0,\Sigma \right)$ with mean **0** and covariance matrix Σ. We assumed $\Sigma =\mathrm{diag}\left(\sigma_{\mu }^{2},\sigma_{f}^{2},\sigma_{g}^{2},\sigma_{fg}^{2}\right)\in {\mathbb{R}}^{4\times 4}$ was a diagonal matrix with positive entries, which means that the individual components were assumed to be independent. The random effects *u*_*i*_ are specific to each network configuration *i*, however we assumed them to be identically distributed. The estimates for $\sigma_{\mu }^{2},\sigma_{f}^{2},\sigma_{g}^{2},\sigma_{fg}^{2}$ quantify the variance of *Z* as a stochastic function of parameter values *w*_*f*_ and *w*_*g*_, considering the stochasticity from random network connectivities. The stochasticity of the INEXA model as well as the residual error was captured in $\varepsilon_{ij}\sim N\left(0,\sigma^{2}\right)$, which we assumed to be normally distributed with mean 0, equal variance σ^2^ and independent of *u*_*i*_ for all indices *i*.

We performed 10 simulations with the INEXA model for each of the nine networks described in section 2.2 and every combination of the parameter values *w*_*f*_, *w*_*g*_ ∈ {1, 1/2, 1/4}. The corresponding values for Ω_*f*_, Ω_*g*_ are listed in Table 1. Fitting this data in *R*, we reported the REML estimates and CIs for $\mu ,\beta_{f},\beta_{g},\beta_{f^{2}},\beta_{g^{2}},\beta_{fg},\Sigma$, and σ^2^. We created contour plots for the response *Z*(*w*_*f*_, *w*_*g*_) as a function of *w*_*f*_ and *w*_*g*_ using the estimates provided by the LME model fit and setting all stochastic terms equal to 0.

#### 2.4.4. Hypothesis 4: Changes in the Excitatory and Inhibitory Synaptic Weights

In this set of simulated experiments, we tested the dependency of neuronal and astrocytic activity on the inhibitory and excitatory synaptic weight boundaries $Y_{\max}^{-}$ and $Y_{\max}^{+}$ (see section 2.1). In our statistical LME model, we considered first and second order fixed effects of synaptic weight boundaries on the response variables as well as a random effect term to account for the variation caused by different network configurations. For ease of notation, let *y*_**in**_ and *y*_**ex**_ denote the parameter values for $Y_{\max}^{-}$ and $Y_{\max}^{+}$, respectively. For every response variable *Z* = *Z*(*y*_**in**_, *y*_**ex**_), we assumed the statistical model

(5) $$\begin{array}{l} Z_{ij}=\mu +\beta_{\mathrm{in}}y_{\text{in}}+\beta_{\mathrm{ex}}y_{\text{ex}}+\beta_{{\mathrm{in}}^{2}}y_{\text{in}}^{2}+\beta_{{\mathrm{ex}}^{2}}y_{\text{ex}}^{2}+\beta_{\mathrm{in},\mathrm{ex}}y_{\text{in}}y_{\text{ex}}\\ \;+\gamma_{i}+\varepsilon_{ij}, \end{array}$$

where the index *i* enumerates the nine network configurations described in section 2.2 and *j* ∈ {1, …, 10} denotes 10 independent repetitions. The variable μ refers to the estimated mean of *Z* if *y*_in_ and *y*_ex_ were equal to zero. The coefficients $\beta_{\mathrm{in}},\beta_{\mathrm{ex}},\beta_{{\mathrm{in}}^{2}},\beta_{{\mathrm{ex}}^{2}},\beta_{\mathrm{in},\mathrm{ex}}\in \mathbb{R}$ correspond to the average increase of *Z* in dependence of the parameters they are multiplied by. Random effects were captured in the random variables γ_*i*_, which we assumed to be independent with zero-mean Gaussian distribution and equal variance $\sigma_{\mathrm{net}}^{2}$. The variance term $\sigma_{\mathrm{net}}^{2}$ quantifies how much the response varies between different stochastically chosen network connectivities if all other parameters are held constant. The residual error was captured in ε_*ij*_, which we assumed to be normally distributed with mean 0, equal variance σ^2^ and independent of γ_*i*_ for all indices *i*.

We performed 10 simulations with the INEXA model for each of the nine networks described in section 2.2 and every combination of the parameter values for $Y_{\max}^{-},Y_{\max}^{+}$ listed in Table 1. Fitting this data in *R*, we reported the REML estimates and 95% CIs for $\mu ,\beta_{\mathrm{in}},\beta_{\mathrm{ex}},\beta_{{\mathrm{in}}^{2}},\beta_{{\mathrm{ex}}^{2}},\beta_{\mathrm{in},\mathrm{ex}},\sigma_{\mathrm{net}}$ and σ. Moreover, we created contour plots for the response *Z*(*y*_**in**_, *y*_**ex**_) as a function of *y*_**in**_ and *y*_**ex**_ using the estimates provided by the LME model fit while setting all stochastic terms equal to 0.

## 3. Results

### 3.1. Hypothesis 1: Astrocyte or Neuron Removal Leads to Reduction of the Network Activity

**Figure 2:**
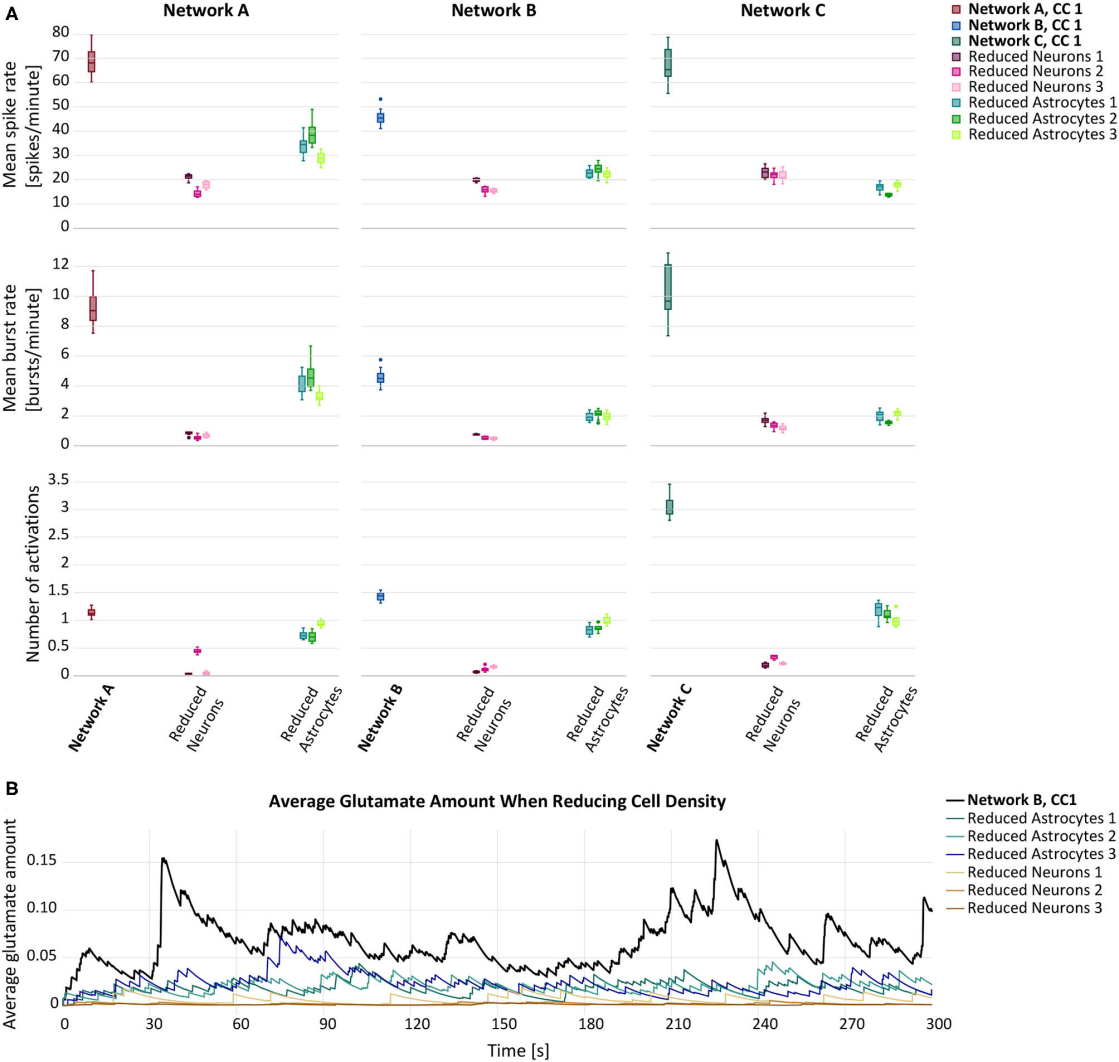
**(A)** Mean spike rate (top), mean burst rate (middle) and mean number of activations per astrocyte (bottom) resulting from the networks with reduced astrocyte or neuron density compared to the default networks A–C with connectivity configuration CC 1 (bold). **(B)** Average glutamate amount in astrocytes when randomly removing 25% of either astrocytes (blue lines) or neurons (brown lines) from a premade network configuration B,CC 1 (black line).

In our first set of simulations, we reduced the cell density of either astrocytes or neurons by 25%. The cell removal resulted in a general decrease of mean spike rate, mean burst rate and mean number of astrocyte activations (Figure 2A). Overall, the decline of the response variables was larger when the neurons were removed from the network. In Figure 2B, the average amount of glutamate prevalent in astrocytes is shown at each time step. While both forms of reduction caused a decrease in astrocytic glutamate, the effect was more pronounced in the case of neuron removal. The *p*-values for the null-hypothesis that the mean response of reduced and default networks coincide were smaller than 10^−10^ for all response variables and both simulated experiments.

The results of the LME model fit are listed in Table 3 for the astrocyte removal and in Table 3 for the neuron removal. They show that cell removal leads to statistically significant activity reduction. The 95% CIs of δ_rem_ are tighter for the neuron removal than for astrocyte removal, indicating that the neuron removal data was fit more accurately by the LME model. The results highlight stark differences between the inter-network standard deviations $\sigma_{\mathrm{rem}}/\hat{\mu }$ of the transformed responses φ(*Z*). While in the astrocyte removal experiments, the inter-network variance of the logarithm of the mean spike rate is relatively low, the standard deviation term for the logarithm of the mean number of astrocyte activations is estimated to be at around 50%. This shows that the robustness to changing network configurations varies significantly for the different response variables considered. Similarly, the standard deviation $\sigma /\hat{\mu }$ of the residual error varies considerably between different response variables, but is consistently smaller than the inter-network standard deviation $\sigma_{\mathrm{rem}}/\hat{\mu }$.

**Table 3:** Estimates and 95% confidence intervals for the quantities estimated in the astrocyte (upper part) and neuron (lower) removal experiments in percent.

	**Mean spike rate**			**Mean burst rate**			**Mean # astrocyte activations**		
**Transformation**	**φ(·) = log(·)**			**φ(·) = log(·)**			**φ(·) = log(·)**		
**Parameter**	**Est**	**CI_**2.5**_**	**CI_**97.5**_**	**Est**	**CI_**2.5**_**	**CI_**97.5**_**	**Est**	**CI_**2.5**_**	**CI_**97.5**_**
**ASTROCYTE REMOVAL**									
${\hat{\delta }}_{\text{rem}}\cdot 100\%$	65.90	56.50	73.27	72.45	59.94	81.05	53.96	41.33	63.87
${\hat{\sigma }}_{\mathrm{rem}}/\hat{\mu }\cdot 100\%$	7.66	5.80	9.29	22.73	17.23	27.57	46.80	35.51	56.74
$\hat{\sigma }/\hat{\mu }\cdot 100\%$	2.42	2.25	2.62	7.29	6.77	7.89	13.02	12.08	14.09
**NEURON REMOVAL**									
${\hat{\delta }}_{\text{rem}}\cdot 100\%$	72.07	65.88	77.14	91.04	86.91	93.87	93.27	86.63	97.60
${\hat{\sigma }}_{\text{rem}}/\hat{\mu }\cdot 100\%$	6.30	4.77	7.64	23.03	17.46	27.92	28.73	21.82	34.81
$\hat{\sigma }/\hat{\mu }\cdot 100\%$	1.94	1.80	2.10	7.11	6.60	7.70	6.00	5.57	6.49

### 3.2. Hypothesis 2: Effect of Astrocytic ATP

**Figure 3:**
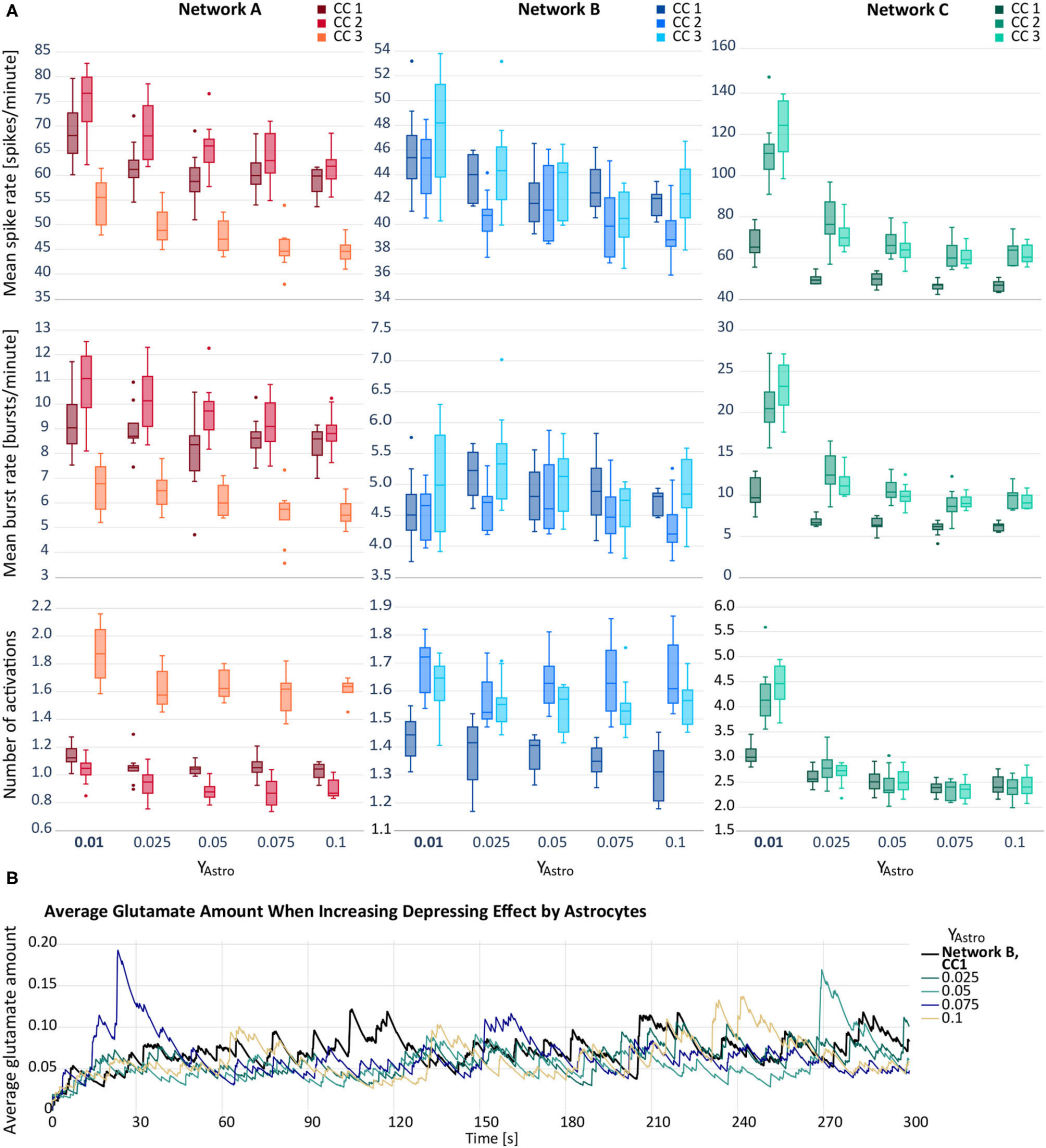
**(A)** Mean spike rate (top), mean burst rate (middle) and mean number of activations per astrocyte (bottom) resulting from the networks with increased astrocytic depression compared to the default networks A–C with *y*_Astro_ = 0.01 (bold). Note that the *y*-axes differ for each subfigure. **(B)** Average glutamate amount in astrocytes when applying a depressive effect on the postsynapse by releasing ATP from the astrocyte. The black line resembles the baseline activity from network B, CC 1. The remaining lines represent deviating values of *y*_Astro_.

For our second hypothesis, we increased the depressing effect of the astrocytic gliotransmitter ATP on excitatory postsynapses. As can be seen in Figure 3A, the depressing effect results in a decreased mean spike rate. In networks A and C, the activity reduction can also be observed for the other two response variables, mean burst rate and astrocyte activity. The increase of the depression, *y*_Astro_, from 0.01 to 0.025 has the proportionally largest effect. In network B, the behaviour of the mean burst rate and the astrocyte activity follows no clear tendency. Figure 3B depicts the average amount of astrocytic glutamate ready for release in presence with an ATP release from the astrocyte. Compared to the baseline level, we observed temporary higher levels of glutamate when amplifying the depressing effect.

In Table 4, we listed the REML estimates and confidence intervals of the parameters of the LME model described in section 2.4.2. We observed a positive second order fixed effect (${\hat{\beta }}_{a^{2}}$) and a negative first-order one (${\hat{\beta }}_{a}$) for all logarithmically transformed responses log(*Z*_*ij*_). While we obtain tight confidence intervals for the baseline activity $\hat{\mu }$, the first and second order coefficients ${\hat{\beta }}_{a},{\hat{\beta }}_{a^{2}}$ have comparatively wider confidence intervals. The standard deviation term ${\hat{\sigma }}_{\mu }$ of log(*Z*_*ij*_) associated with μ is considerably lower than the standard deviation term ${\hat{\sigma }}_{a}$ associated with ${\hat{\beta }}_{a}$, moreover the confidence intervals of ${\hat{\sigma }}_{\mu }$ are narrower than those of ${\hat{\sigma }}_{a}$, indicating that the first order term is difficult to fit by our model. The error standard deviation $\hat{\sigma }$ is comparatively low and has tight confidence intervals for all responses.

**Table 4:** Restricted maximum likelihood estimates, lower and upper ends of 95% confidence intervals for the astrocyte depressing effects experiment.

	**Mean spike rate**			**Mean burst rate**			**Mean # astrocyte activations**		
**Transformation**	**log(·)**			**log(·)**			**log(·)**		
**Parameter**	**Est**	**CI_**2.5**_**	**CI_**97.5**_**	**Est**	**CI_**2.5**_**	**CI_**97.5**_**	**Est**	**CI_**2.5**_**	**CI_**97.5**_**
${\hat{\sigma }}_{\mu }$	0.31	0.20	0.51	0.51	0.32	0.82	0.48	0.30	0.78
${\hat{\sigma }}_{a}$	1.90	1.15	3.13	2.98	1.81	4.92	1.96	1.19	3.23
$\hat{\sigma }$	0.10	0.09	0.11	0.15	0.14	0.16	0.10	0.09	0.11
$\hat{\mu }$	4.25	4.03	4.46	2.24	1.89	2.59	0.72	0.39	1.05
${\hat{\beta }}_{a}$	–8.92	–10.67	–7.16	–9.39	–12.13	–6.64	–7.67	–9.47	–5.88
${\hat{\beta }}_{a^{2}}$	57.12	46.01	68.23	57.93	40.66	75.20	52.53	41.34	63.73

### 3.3. Hypothesis 3: Reducing the Glutamate Release and Uptake Rate Results in Higher Network Activity

**Figure 4:**
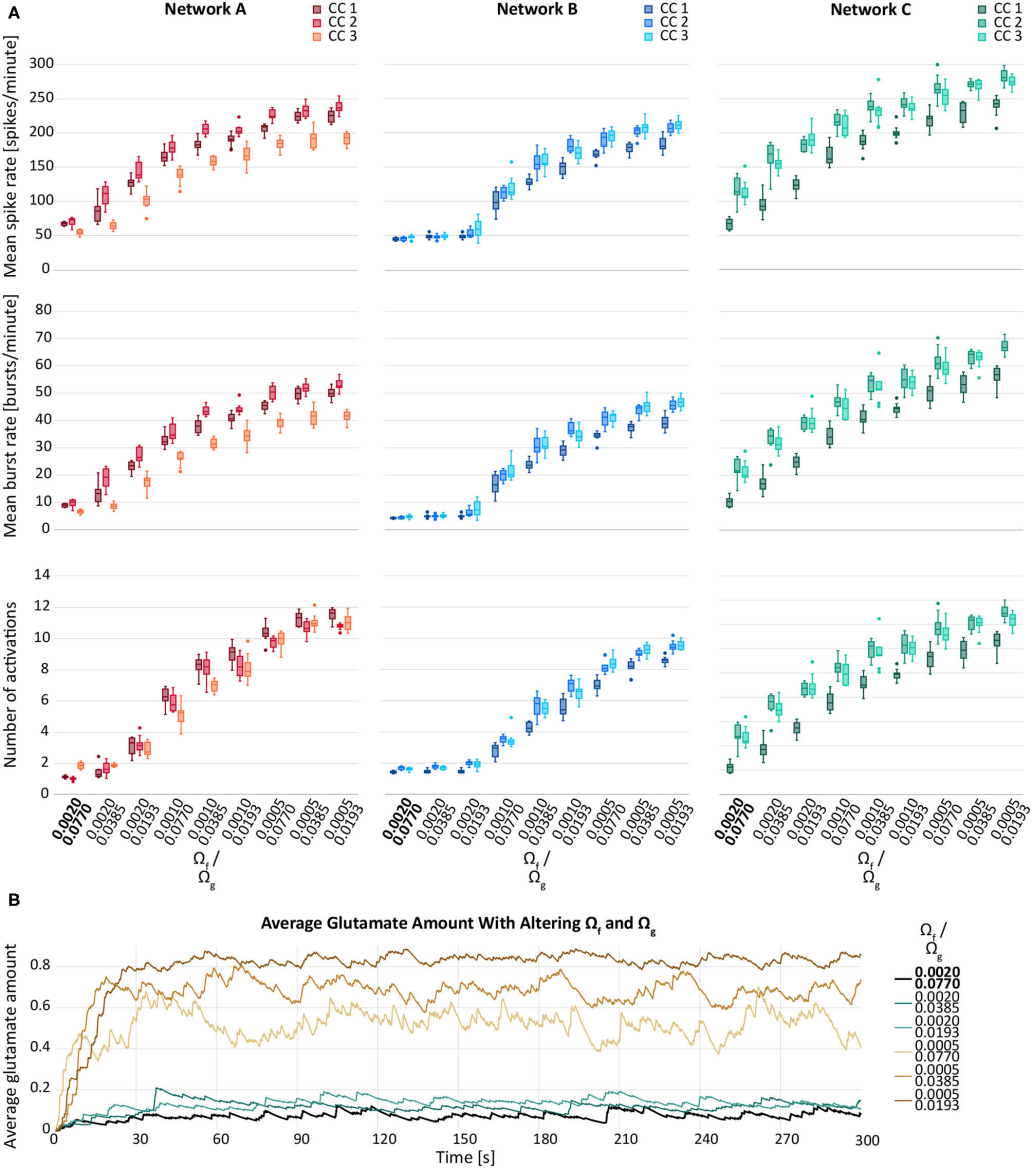
**(A)** Mean spike rate (top), mean burst rate (middle) and mean number of activations per astrocyte (bottom) for varying rates of synaptic facilitation, Ω_*g*_, and varying recovery rates of the gliotransmitter receptors, Ω_*g*_, for networks A–C and their connectivity configurations CC 1, 2, 3. The default values are indicated in bold. **(B)** Average glutamate amount in astrocytes when varying the synaptic facilitation rate, Ω_*g*_, and the recovery rate of the gliotransmitter receptors, Ω_*g*_. The black line resembles the baseline activity from network B, CC 1. The brown lines represent the changes by Ω_*f*_ and the blue lines the changes by Ω_*g*_.

Figure 4A depicts the behaviour of the response variables with respect to varying rates of synaptic facilitation, Ω_*f*_, and varying recovery rates of the gliotransmitter receptors, Ω_*g*_. In general, a reduction in both, Ω_*g*_ and Ω_*f*_, resulted in an elevation of the response variables. However, the increase between two consecutive values for Ω_*f*_ was larger than between two values of Ω_*g*_. An exception was observed for network B, for which the modification of Ω_*g*_ did not result in any considerable changes of the response variables.

We observed a similar behaviour for the average glutamate level when varying Ω_*f*_ and Ω_*g*_. Figure 4B shows a larger effect of Ω_*g*_ compared to the effect of Ω_*f*_. Decreasing either parameter resulted in elevated response variables compared to the default network. Also in this case, the effect of Ω_*f*_ was larger compared the effect of Ω_*g*_.

The results of the LME model fit are reported in Table 5. We observed that the average response μ fitted for values *w*_*f*_ = *w*_*g*_ = 0 (baseline activity) was difficult to estimate, indicated by the wide confidence intervals. Also the estimated standard deviation ${\hat{\sigma }}_{\mu }$ of the baseline activity due to different network configurations was large relative to μ for all response variables, particularly for the mean number of astrocyte activations. For most response variables, the estimated standard deviation $\hat{\sigma }$ of the residual error was at around 10% of the baseline activity, significantly smaller than ${\hat{\sigma }}_{\mu }$.

**Table 5:** Restricted maximum likelihood estimates, lower and upper ends of 95% confidence intervals for the glutamate dynamics experiment.

	**Mean spike rate**			**Mean burst rate**			**Mean # astrocyte activations**		
**Parameter**	**Est**	**CI_**2.5**_**	**CI_**97.5**_**	**Est**	**CI_**2.5**_**	**CI_**97.5**_**	**Est**	**CI_**2.5**_**	**CI_**97.5**_**
${\hat{\sigma }}_{\mu }$	24.97	15.31	41.57	7.57	4.70	12.57	1.14	0.69	1.90
${\hat{\sigma }}_{f}$	45.08	27.13	73.45	9.84	5.80	16.07	2.61	1.60	4.25
${\hat{\sigma }}_{g}$	23.38	12.57	39.18	5.61	2.89	9.44	1.39	0.80	2.31
${\hat{\sigma }}_{fg}$	50.91	29.52	83.58	12.59	7.19	20.68	2.55	1.50	4.19
$\hat{\sigma }$	12.50	11.88	13.14	3.23	3.07	3.40	0.62	0.59	0.65
$\hat{\mu }$	281.19	262.43	299.95	68.72	63.14	74.30	15.26	14.39	16.12
${\hat{\beta }}_{f}$	–151.58	–187.47	–115.69	–53.21	–61.46	–44.96	–16.12	–18.13	–14.12
${\hat{\beta }}_{g}$	–41.36	–66.23	–16.49	–13.86	–20.11	–7.61	–2.82	–4.15	–1.48
${\hat{\beta }}_{f^{2}}$	3.64	–11.23	18.52	10.72	6.87	14.57	5.45	4.71	6.18
${\hat{\beta }}_{g^{2}}$	18.60	3.73	33.48	6.05	2.20	9.90	0.59	–0.15	1.32
${\hat{\beta }}_{fg}$	–43.82	–79.56	–8.07	–8.97	–17.82	–0.12	–0.29	–2.08	1.51

**Figure 5:**
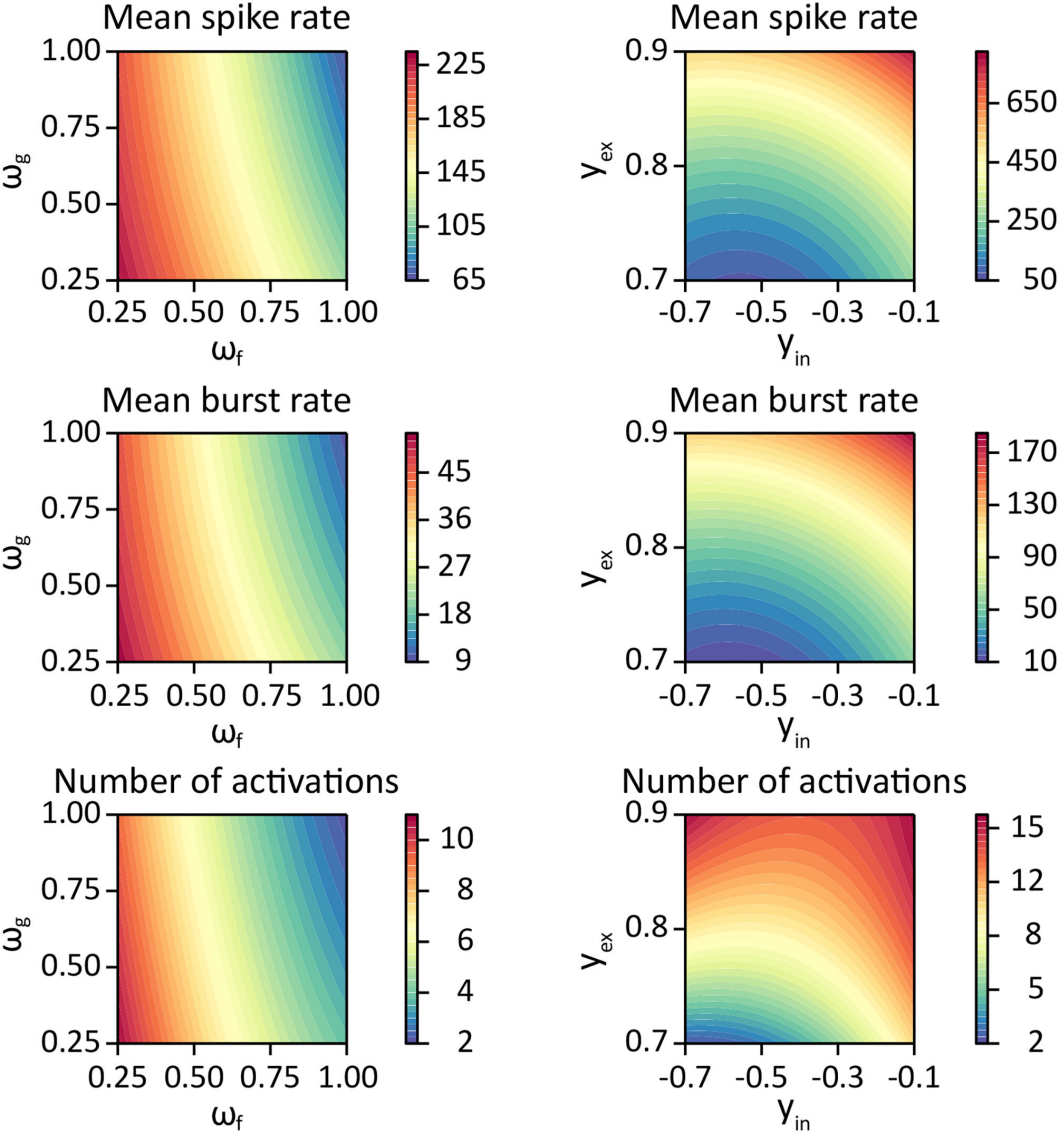
Contour plots for each response variable as a function of glutamate dynamics parameters ω_*f*_, ω_*g*_ (left column) using parameter estimates listed in Table 5, and as a function of the synaptic weight bound parameters *y*_**in**_, *y*_**ex**_ (right column) using the parameter estimates from Table 6.

The parameters $\beta_{f},\beta_{f^{2}}$ had tight confidence intervals, indicating that the estimates were reliable. We saw a strong negative first order effect of the rate of presynaptic glutamate uptake *w*_*f*_ on all responses and a positive second-order effect. For all responses, the standard deviation estimate σ_*f*_ of the first order effect was small relative to ${\hat{\beta }}_{f}$. Similarly, we found a negative first order and a positive second-order effect of the astrocyte glutamate uptake on all variables with small estimated standard deviation ${\hat{\sigma }}_{g}$. The corresponding 95% CIs for the parameters $\beta_{g},\beta_{g^{2}}$ were slightly wider than those of β_*f*_ and $\beta_{f^{2}}$. The first-order interaction coefficient β_*fg*_ of the parameters *w*_*f*_ and *w*_*g*_ was significant, but with a small effect for all responses. The fact that ${\hat{\sigma }}_{fg}$ and β_*fg*_ lay in a similar range indicates that the interaction terms might vary considerably for different network configurations. However, these effects were all small compared to β_*f*_ or β_*g*_. Contour plots of the response variables derived from the LME model fit are shown in Figure 5.

### 3.4. Hypothesis 4: Higher Network Activity When Increasing Synaptic Weight Parameters

**Figure 6:**
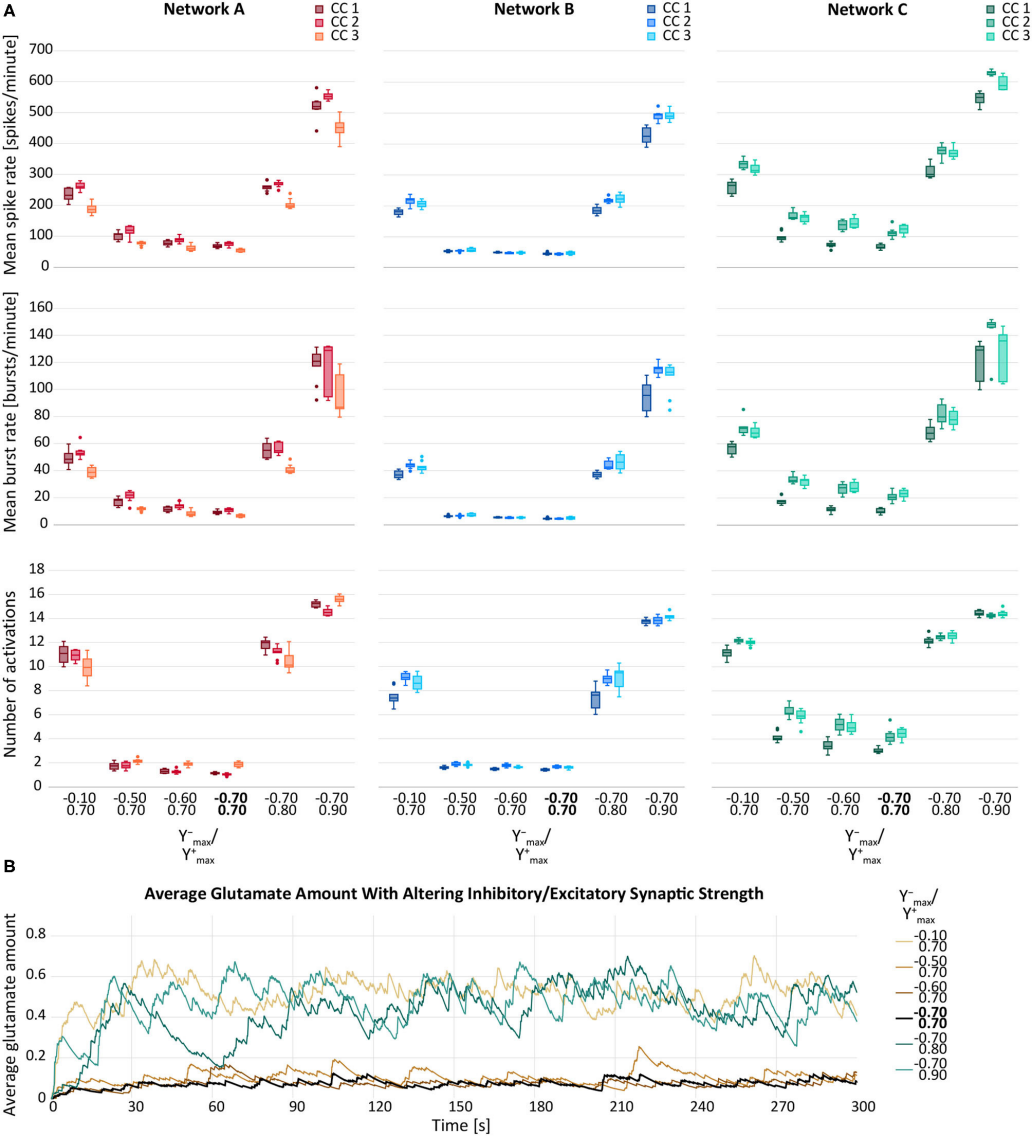
**(A)** Mean spike rate (top), mean burst rate (middle) and mean number of activations per astrocyte (bottom) for varying maximum inhibitory ($Y_{max}^{-}$) and maximum excitatory synaptic weight ($Y_{max}^{+}$) of neurons for networks A–C and their connectivity configurations CC 1, 2, 3. The default values are indicated in bold. **(B)** Average glutamate amount in astrocytes when altering the inhibitory and excitatory synaptic weights. The black line resembled the activity from the default network B, CC 1. The brown lines represent the changes of $Y_{max}^{-}$ and the blue lines the changes in $Y_{max}^{+}$.

In this section, we computationally investigated the neuronal and astrocytic response to synaptic perturbations caused by SCZ. Figure 6A depicts the behaviour of the three response variables while changing either the maximum inhibitory synaptic weight $Y_{max}^{-}$ or in the maximum excitatory synaptic weight $Y_{max}^{+}$. Increases in $Y_{max}^{-}$ and $Y_{max}^{+}$ yielded in elevated response variables. The relationship between the parameter distortion and the effect on the observation variables were consistent among the three networks (A, B, C) and their corresponding connectivities (1, 2, 3). However, network B showed a comparatively reduced response to deviations in $Y_{max}^{-}$. The variance induced by alterations of $Y_{max}^{+}$ was higher than the inter-network and the inter-connectivity variance. This was not the case for the variance induced by deviating $Y_{max}^{-}$, which was due the lower number of inhibitory cells in the networks. Moreover, compared to the unmodified parameter configuration ($Y_{max}^{-}=-0.7$ and $Y_{max}^{+}=0.7$), the response variables from the distorted parameter configurations showed higher variances in most of the cases.

In Figure 6B, the average glutamate amount in all astrocytes is plotted against time for varying $Y_{max}^{-}$ and $Y_{max}^{+}$. We noted that a change in the glutamate behaviour was achieved by all deviation levels of $Y_{max}^{+}$. For $Y_{max}^{-}$, a large deviation from the default values was required to see a change in the average glutamate levels. The smaller deviations of $Y_{max}^{-}$ did not yield a notable elevation of neither the response variables (Figure 6A) nor the time course of the average glutamate level (Figure 6B).

The parameter estimates of the corresponding statistical LME analysis are listed in Table 6. We refer to the average μ of the response variable when setting parameter values *y*_in_, *y*_ex_ to zero as the baseline activity. Comparing the parameter estimates in Table 6 to the data in Figure 6A, we observe that the estimated baseline activity $\hat{\mu }$ is significantly larger than the average response. This effect is compensated by large values of parameters ${\hat{\beta }}_{\mathrm{in}}$,${\hat{\beta }}_{\mathrm{ex}}$,${\hat{\beta }}_{{\mathrm{in}}^{2}}$,${\hat{\beta }}_{{\mathrm{ex}}^{2}}$ and ${\hat{\beta }}_{\mathrm{in},\mathrm{ex}}$. Thus, to analyse the magnitude of the standard deviation terms, we refer to Figure 6A. We observed that the estimated residual standard deviation $\hat{\sigma }$ was at around 1/10 to 1/2 of the average response value. The confidence intervals for σ_net_ were wide, so the random effect due to different network connectivities was not clear. However, across all experiments, ${\hat{\sigma }}_{\mathrm{net}}$ was estimated to be smaller than $\hat{\sigma }$. Thus, it is likely that on average, responses vary more significantly for different repetitions than between networks with different connectivities. Further, we observed relatively wide confidence intervals for all fitted coefficients, indicating that the model fit and the provided estimates have limited expressiveness. Contour plots of the response variables derived from the LME model fit are shown in Figure 5.

**Table 6:** Restricted maximum likelihood estimates, lower and upper ends of 95% confidence intervals for the synaptic weight parameter experiments.

	**Mean spike rate**			**Mean burst rate**			**Mean # astrocyte activations**.		
**Par**	**Est**	**CI_**2.5**_**	**CI_**97.5**_**	**Est**	**CI_**2.5**_**	**CI_**97.5**_**	**Est**	**CI_**2.5**_**	**CI_**97.5**_**
${\hat{\sigma }}_{\mathrm{net}}$	38.49	23.96	63.00	7.92	4.87	13.02	0.62	0.36	1.03
$\hat{\sigma }$	58.89	56.35	61.33	16.42	15.71	17.10	2.07	1.98	2.15
$\hat{\mu }$	2607.76	2129.18	3086.35	582.85	449.47	716.23	–64.05	–80.83	–47.27
${\hat{\beta }}_{\mathrm{in}}$	533.64	361.76	705.52	122.23	74.30	170.15	79.58	73.55	85.61
${\hat{\beta }}_{\mathrm{ex}}$	–7845.90	–9040.42	–6651.38	–1783.96	–2117.05	–1450.86	182.21	140.29	224.14
${\hat{\beta }}_{{\mathrm{in}}^{2}}$	7456.40	6628.56	8284.23	1582.80	1351.95	1813.64	183.75	154.69	212.80
${\hat{\beta }}_{{\mathrm{ex}}^{2}}$	6623.51	5879.52	7367.50	1519.87	1312.40	1727.33	–102.41	–128.52	–76.29
${\hat{\beta }}_{\mathrm{in},\mathrm{ex}}$	751.31	562.72	939.90	163.00	110.41	215.59	–67.54	–74.16	–60.92

## 4. Discussion

Schizophrenia is characterised by diverse dysregulations and malfunctions in neurons and astrocytes (Takahashi and Sakurai, 2013; Moraga-Amaro et al., 2014; Mei et al., 2018). In astrocytes, the pathology is among others indicated by a higher GFAP expression, an altered morphology, loss of gap junctions between cells, and an elevated glutamate release (Mitterauer, 2009; Catts et al., 2014; Mei et al., 2018). In neurons, SCZ is indicated by morphological alterations, reductions in cell density, and excessive glutamate release, which is due to the hypofunction of NMDA receptors (Cotter et al., 2001; Mei et al., 2018). Spontaneous neuronal activity is a fine balance between excitation and inhibition (Lisman, 2012; Sohal and Rubenstein, 2019), which is between 30 and 200 spikes per minute in healthy human stem cell-derived neurons (Tukker et al., 2018; Kizner et al., 2019). However, it is disputed whether SCZ causes an increase or a decrease in neuronal and astrocytic activity (Mei et al., 2018; Naujock et al., 2020).

Computational models can support the understanding of biophysical pathways when experiments are not realisable or too expensive. So far, a multitude of biophysical (bottom-up) and symptom focused (top-down) models have been developed. According to the reviews by Rolls et al. (2008) and Valton et al. (2017), top-down models mostly support the *dopamine hypothesis*. For example, Hoffman and McGlashan (2006) explored the effects of neuronal pruning and excessive dopamine. Using a recurrent backpropagation model of working memory, they showed how neuron reduction or hyperdopamigenic systems can produce spontaneous percepts simulating hallucinated speech. Furthermore, they were able provide an explanation for the onset of SCZ in late adolescence. For the *glutamate hypothesis*, only a few computational models are available (Valton et al., 2017). The models mostly focused on excitatory and inhibitory neurons, showing that deficits in glutamatergic or GABAergic activity lead to impairments in working-memory and other cognitive processing (Valton et al., 2017).

In the last few years, attempts were made to simulate SCZ using the Bayesian inference hypothesis. This hypothesis assumes that the brain combines sensory evidence together with prior knowledge and expectation to interpret a stimulus. In SCZ, different delusions bias the expected value, leading to stronger perceptual biases in the future. So far, the hypothesis has mostly been tested for illusions (Valton et al., 2017). One of the first computational models in that area was made by Adams et al. (2013). Using a biological predictive coding scheme, they showed that psychosis in SCZ can be accounted by an increased prior precision and trait abnormalities (e.g., abnormalities of smooth pursuit eye movements, event-related brain potentials or anhedonia) by a decrease in prior precision.

The loss of gap junctions in astrocytes can be a hallmark in SCZ (Mitterauer, 2009). Using the INEXA model, Genocchi et al. (2020) studied the effect of gap junction uncoupling on the neuronal activity. Additionally, Lenk et al. (2021) showed that larger distances between astrocytes, and thus, less gap junctions, seem to centralise the information transmission in astrocytes. The spike rate and burst rate increased until a cell-cell distance of 100 μm and then decreased again.

In this paper, we investigated the effect resulting from four different hypotheses of SCZ on neuronal and astrocytic activity. Using the computational network model by Lenk et al. (2020), we separately examined the neuronal and astrocytic dynamics depending on (1) the neuron and astrocyte density in the network, (2) the depressing effect of astrocytes on excitatory neurons, (3) the glutamate release from the presynapse and the glutamate uptake by the adjacent astrocyte, and (4) the excitatory and/or inhibitory synaptic weight between neurons. Furthermore, we applied a set of statistical models to quantify the effect of these pathological changes.

Several studies report that the neuronal and astrocytic density decreases in several brain regions in SCZ (Cotter et al., 2001; Brennand et al., 2011; Williams et al., 2013), which may be caused by excessive glutamate levels (Drouin-Ouellet et al., 2011). Wagenaar et al. (2006) studied the effect of neuronal cell density on the spike and burst behaviour recorded with MEAs. They found that sparser cultures exhibit a lower spike rate as well as delayed network development. In our simulated experiments, we found that reducing the number of neurons or astrocytes by 25% (hypothesis (1)) causes a significant decrease in neuronal and astrocytic activity. In the original paper of our computational model (Lenk et al., 2020), the number of astrocytes was increased, which led to an elevation of the neuronal activity. Conversely, this means that when astrocytes and their connections are eliminated, neuronal activity also decreases. Astrocytes are assumed to have a homeostatic effect on neurons, they can both enhance and dampen neuronal activity (Santello et al., 2019). Additionally, we found that the effect of reducing astrocytes or neurons can vary significantly for different network connectivity configurations. While the effect on the mean spike rate is robust to varying network connectivity configurations, the mean burst rate and the number of astrocyte activations are not. This might be useful for relating the behaviours of networks with the same number of cells, but different or unknown network configurations.

Furthermore, we have studied the effect of ATP release from the astrocyte on the postsynapse [hypothesis (2)]. Lalo et al. (2016) have demonstrated that ATP activates postsynaptic P2XRs resulting in a downregulation of neuronal NMDARs. This negative feedback mechanism might prevent cell damage due to excessive Ca^2+^ and glutamate concentrations (Singh and Abraham, 2017). By the regulation of NMDARs, P2XRs might also be involved in synaptic plasticity and long-term potentiation (LTP) (Moraga-Amaro et al., 2014; Lalo et al., 2016; Singh and Abraham, 2017). This receptor type could even have a protective role by avoiding excitotoxicity and excessive LTP (Lalo et al., 2016).

An increase in neuronal and astrocytic activity was observed when altering the rate of presynaptic facilitation and the recovery rate of mGluRs [hypothesis (3)]. The decreased facilitation rate increases the amount of neurotransmitters ready for release to the synaptic cleft, thereby increasing the release probability and spike rate. Similarly, decreasing the recovery rate of gliotransmitter receptors results in a higher presynaptic potentiation (Helen et al., 1992; Agulhon et al., 2008). Subsequently, this leads to an increase of the neuronal activity.

According to the *glutamate hypothesis of SCZ*, NMDARs undergo a hypofunction leading to an excessive glutamate release (Mei et al., 2018). The changes in the NMDAR function may be caused by D-serine (Takahashi and Sakurai, 2013; Cohen et al., 2015; Mei et al., 2018), which we implicitly modelled by increasing the excitatory synaptic weights. In the future, we aim to add D-serine explicitly to the INEXA model for investigating its role in long-term potentiation and thus memory (Henneberger et al., 2010). Furthermore, GABAergic interneurons (Uhlhaas and Singer, 2010) might be impacted by SCZ due to reaction to the alterations in the NMDARs and an impaired synthesis and reuptake of GABA into the cell (Uhlhaas and Singer, 2010; Moghaddam and Javitt, 2012; Mei et al., 2018). When investigating hypothesis (4), the results show a higher neuronal and astrocytic activity, when increasing the inhibitory or excitatory synaptic weights (both yields in a neuronal excitation).

Further experimental observations are coherent with our modelling results. In SCZ, glutamine synthetase, which catalyzes glutamate, was shown to have reduced activity leading to higher levels of intracellular astrocytic glutamate, and thus, result in a neuronal hyperexcitability (Hu et al., 2015; Mei et al., 2018). This observation is in agreement with the results of our simulated experiments from hypotheses (3) and (4). The accumulation of glutamate was most pronounced in simulated experiments with a decreased mGluR recovery rate. In these experiments, glutamate accumulated quickly before stabilizing at an extremely high level. As to be expected, the neuronal activity increased with dysinhibition of the GABAergic neurons or the excitation of the glutamatergic neurons.

In the INEXA model, we mainly concentrated on the IP_3_-dependent Ca^2+^ dynamics. Srinivasan et al. (2015) showed that somatic calcium is abolished in adult Ip3r2^−/−^ mice. In the future, we intend to extend the model by adding astrocytic NMDARs and α-amino-3-hydroxy-5-methyl-4-isoxazolepropionic acid (AMPA) receptors as well as the glutamate transporter-dependent pathway to gain further insights into the possible dysfunctions in schizophrenia.

Dynamical causal modeling (DCM), a framework using Bayesian statistics to compare competing models (Friston et al., 2003), has given rise to differential diagnosis methods for psychiatric diseases (Stephan et al., 2017). In the future, we will apply Bayesian inference to obtain posterior distributions of certain astrocytic parameters from INEXA, building the basis for Bayesian model selection among competing disease mechanisms linked to astrocyte malfunction.

## Data Availability Statement

The datasets for this study can be found in Github: https://github.com/kerstinlenk/INEXA_SCZ/.

## Author Contributions

KL wrote the first draft of the manuscript. LF, JL, and FS collected simulation data. LF, FS, and KL visualized the results. FS designed and performed the statistical analysis. All authors designed and performed research and contributed to the manuscript writing and revision. In addition, they have read and approved the submitted version.

## Conflict of Interest

The authors declare that the research was conducted in the absence of any commercial or financial relationships that could be construed as a potential conflict of interest.

## Publisher's Note

All claims expressed in this article are solely those of the authors and do not necessarily represent those of their affiliated organizations, or those of the publisher, the editors and the reviewers. Any product that may be evaluated in this article, or claim that may be made by its manufacturer, is not guaranteed or endorsed by the publisher.
