# Supplementary material for: Neuronal and Astrocytic Regulations in Schizophrenia: A Computational Modelling Study
Source: Front Cell Neurosci. 2021 Aug 26;15:718459. doi: 10.3389/fncel.2021.718459 (PMC8428975; doi:10.3389/fncel.2021.718459)
Supplement: Supplementary file 1 [file Data_Sheet_1.PDF]

Supplementary Material

CELL REMOVAL EXPERIMENTS

1 ANALYSING CELL REMOVAL WITH LINEAR MIXED EFFECTS MODELS

In the cell removal experiment we wish to quantify fixed effects of cell removal and random effects of different network configurations on astrocyte and neuron response variables. Let $d_a, d_n \in \{0,1,2,3\}$ denote categorical variables for astrocyte, respectively neuron removal. Since the mathematical models for these experiments are identical, we ignore the subscript for cell type and simply write d for the rest of the description. The variable $d = 0$ corresponds to keeping all cells, while $d = k$ corresponds to a cell removal of 25% in the kth copy of a network for $k \in \{1,2,3\}$. Let us denote the indicator function by

$$\mathbb{1}(x) = \begin{cases} 1 & \text{statement } x \text{ holds} \\ 0 & \text{otherwise.} \end{cases} \tag{S1}$$

For every response variable Z, we choose a transformation $\varphi : \mathbb{R} \to \mathbb{R}$ and assume the LME model

$$\varphi(Z_{ij}) = \mu \cdot \mathbb{1}(d=0) + \beta_1 \cdot \mathbb{1}(d=1) + \beta_2 \cdot \mathbb{1}(d=2) + \beta_3 \cdot \mathbb{1}(d=3) + \gamma_i + \varepsilon_{ij}. \tag{S2}$$

The index i enumerates the network configuration described in Section 3.2, j the simulation repetitions. The variable μ refers to the mean of the transformed response $\varphi(Z)$ before cell removal, $\beta_1, \beta_2, \beta_3 \in \mathbb{R}$ are coefficients to be fitted. The random variables $\gamma_i \sim \mathcal{N}(0, \sigma^2_{\text{rem}})$ are independent and normally distributed, with common variance σ^2_{rem}, corresponding to the variability caused by different network connectivity configurations. Finally, $\varepsilon_{ij} \sim \mathcal{N}(0, \sigma^2)$ are independent Gaussian error terms capturing the residual error.

We performed ten simulations with the INEXA model for each of the 36 networks described in Section 3.2. Fitting this data in R, we report the restricted maximum likelihood (REML) estimates and 95% confidence intervals (CIs) for $\mu, \beta_1, \beta_2, \beta_3, \sigma_{\text{rem}}$ and σ.

To determine the difference in response means between keeping and dropping cells, we use contrasts (Oehlert, 2010), testing for the null hypothesis

$$H_0 : \Delta := \tfrac{1}{3}(\beta_1 + \beta_2 + \beta_3) = 0. \tag{S3}$$

The fit returns an REML estimate and 95% CIs for Δ. It also returns the p-value of this null-hypothesis, which we report in the main text. Tables S1 and S2 summarise the findings from the cell drop experiments. We want to emphasise that these fits describe the transformed reponse variables $\varphi(Z)$. We will address the interpretation of these results in the original scale in the next subsection.

From Tables S1 and S2 we see that the standard deviation of random effects term σ_{rem} which describes the variation of $\varphi(Z)$ given different network configurations is slightly larger than the residual error. Relative to the mean response $\hat{\mu}$, σ_{rem} ranges from being small for the mean spike rate and comparatively large for the other responses. This indicates that the mean spike rate is more robust with resepct to varying network configurations than the mean burst rate and the mean number of astrocyte activations.

The residual errors have narrow CIs, indicating that they can be estimated well by the model. Relative to the estimated mean reponse $\hat{\mu}$ the residual errors are small for mean spike rate in both experiments, while somewhat larger for the other responses.

For both experiments, the coefficients $\beta_l, l = 1, 2, 3$ have similar REML estimates and confidence intervals. A possible interpretation for this is that the fixed effect the model attributes to cell removal is similar for the three copies of a network in which cells were randomly removed. However, to assess the variation of the reponse due to stochastic deletion of cells, one also needs to consider the random effects term σ_{rem}, which has varying relative size for the different response variables.

In all experiments, the average reduction Δ of the transformed response $\varphi(Z)$ is significant.

	Mean Spike Rate			Mean Burst Rate			Mean # Astrocyte Activations		
Transformation	$\varphi(\cdot) = \log(\cdot)$			$\varphi(\cdot) = \log(\cdot)$			$\varphi(\cdot) = \log(\cdot)$		
Parameter	Estimate	$\text{CI}_{2.5}$	$\text{CI}_{97.5}$	Estimate	$\text{CI}_{2.5}$	$\text{CI}_{97.5}$	Estimate	$\text{CI}_{2.5}$	$\text{CI}_{97.5}$
$\hat{\sigma}_{\text{red}}$	0.32	0.24	0.39	0.49	0.37	0.60	0.32	0.24	0.39
$\hat{\sigma}$	0.10	0.09	0.11	0.16	0.15	0.17	0.09	0.08	0.10
$\hat{\mu}$	4.19	3.99	4.40	2.17	1.86	2.49	0.68	0.48	0.89
$\hat{\beta}_1$	-1.05	-1.33	-0.76	-1.24	-1.68	-0.79	-0.83	-1.11	-0.54
$\hat{\beta}_2$	-1.10	-1.39	-0.81	-1.32	-1.77	-0.88	-0.86	-1.15	-0.57
$\hat{\beta}_3$	-1.09	-1.37	-0.80	-1.30	-1.75	-0.86	-0.64	-0.92	-0.35
$\hat{\Delta}$	-1.08	-1.32	-0.83	-1.29	-1.66	-0.91	-0.78	-1.02	-0.53

Table S1. Parameter estimates and 95% confidence intervals for astrocyte removal experiments.

	Mean Spike Rate			Mean Burst Rate			Mean # Astrocyte Activations		
Transformation	$\varphi(\cdot) = \log(\cdot)$			$\varphi(\cdot) = \log(\cdot)$			$\varphi(\cdot) = \text{id}(\cdot)$		
Parameter	Estimate	$\text{CI}_{2.5}$	$\text{CI}_{97.5}$	Estimate	$\text{CI}_{2.5}$	$\text{CI}_{97.5}$	Estimate	$\text{CI}_{2.5}$	$\text{CI}_{97.5}$
$\hat{\sigma}_{\text{red}}$	0.26	0.20	0.32	0.50	0.38	0.61	0.65	0.50	0.79
$\hat{\sigma}$	0.08	0.08	0.09	0.15	0.14	0.17	0.14	0.13	0.15
$\hat{\mu}$	4.19	4.03	4.36	2.17	1.85	2.49	2.27	1.86	2.68
$\hat{\beta}_1$	-1.32	-1.56	-1.08	-2.48	-2.93	-2.03	-2.12	-2.71	-1.54
$\hat{\beta}_2$	-1.26	-1.50	-1.02	-2.34	-2.79	-1.89	-2.11	-2.69	-1.52
$\hat{\beta}_3$	-1.25	-1.48	-1.01	-2.41	-2.86	-1.96	-2.12	-2.71	-1.54
$\hat{\Delta}$	-1.28	-1.48	-1.08	-2.41	-2.79	-2.03	-2.12	-2.61	-1.62

Table S2. Parameter estimates and 95% confidence intervals for neuron removal experiments.

2 INTERPRETATION OF THE RESULTS ON THE ORIGINAL SCALES

So far we have analysed the LME model fitted to transformed response variables $\varphi(Z)$. Here we describe how transformations of the reponse affect the interpretation of the results and how we chose to compute the average reduction of cell activity δ_{rem}. For most response variables we used a logarithmic transformation in equation S2 to obtain approximately normally distributed residuals in the LME model fit, matching the LME model assumptions. With a logarithmic transformation, the reponse is assumed to be of the form

$$Z_{ij} = e^{\mu} e^{\beta_1 \cdot \mathbb{1}(d=1)} e^{\beta_2 \cdot \mathbb{1}(d=2)} e^{\beta_3 \cdot \mathbb{1}(d=3)} e^{\gamma_i} e^{\varepsilon_{ij}}. \tag{S4}$$

Thus the fixed effects coefficients $\mu, \beta_1, \beta_2, \beta_3$ have a multiplicative effect on the reponse Z, which follows a lognormal distribution under this assumption. Since the median is a standard location estimator for

lognormal random variables Limbert et al. (2001), we compare the median reponse of networks with regular cell number to those with reduced cell number. For these responses we define the average reduction of cell activity δ_{rem} as

$$\delta_{\text{rem}} = 1 - \frac{\text{median}(Z \cdot \mathbb{1}(d \neq 0))}{\text{median}(Z \cdot \mathbb{1}(d = 0))}.$$

This can be interpreted as the average factor by which the reponse is reduced upon cell removal. Since for normally distributed random variables, the median coincides with the mean, we have

$$\begin{aligned}
&\text{median}(\log(Z_{ij}) \cdot \mathbb{1}(d \neq 0)) = \mathbb{E}[\log(Z_{ij}) \cdot \mathbb{1}(d \neq 0)] = \mu + \tfrac{1}{3}(\beta_1 + \beta_2 + \beta_3) \quad \text{and} \\
&\text{median}(\log(Z_{ij}) \cdot \mathbb{1}(d = 0)) = \mathbb{E}[\log(Z_{ij}) \cdot \mathbb{1}(d = 0)] = \mu.
\end{aligned}$$

Since the median transforms with strictly increasing, continuous functions Hosseini (2010), the medians of the non-transformed responses are given by

$$\begin{aligned}
&\text{median}(Z \cdot \mathbb{1}(d \neq 0)) = e^{\mu + (\beta_1 + \beta_2 + \beta_3)/3} \quad \text{and} \\
&\text{median}(Z \cdot \mathbb{1}(d = 0)) = e^{\mu}
\end{aligned}$$

Thus the ratio of medians is given by

$$\delta_{\text{rem}} = 1 - \frac{\text{median}(Z \cdot \mathbb{1}(d \neq 0))}{\text{median}(Z \cdot \mathbb{1}(d = 0))} = 1 - e^{(\beta_1 + \beta_2 + \beta_3)/3} = 1 - e^{\Delta}.$$

The LME fit provides restricted maximum likelihood (REML) estimates and 95% confidence intervals (CI) for Δ, which we use to report estimates and 95% CIs for δ_{rem}.

When we applied no transformation to the data, we define the average reduction of cell activity δ_{rem} in relative terms as

$$\delta_{\text{rem}} = -\frac{(\beta_1 + \beta_2 + \beta_3)/3}{\mu} = -\frac{\Delta}{\mu}.$$

that is, the average relative decrease of cell activity upon cell removal. We use estimates and 97.5% CIs for Δ and μ provided by the LME model fit to compute estimates and 95% CIs for δ_{rem}. Concretely, given the REML estimates $\hat{\Delta}$, $\hat{\mu}$ from the fit, we compute the estimate $\hat{\delta}_{\text{rem}} = -\frac{\hat{\Delta}}{\hat{\mu}}$. Given 97.5% confidence intervals

$$C_{\Delta}^{0.975} = [\ell_{\Delta}, u_{\Delta}]$$

for Δ and

$$C_{\mu}^{0.975} = [\ell_{\mu}, u_{\mu}]$$

for μ we compute a 95% confidence interval

$$C_{\Delta/\mu}^{0.95} = [\tfrac{\ell_{\Delta}}{u_{\mu}}, \tfrac{u_{\Delta}}{\ell_{\mu}}] =: [\ell_{\Delta/\mu}, u_{\Delta/\mu}]$$

for Δ/μ. The corresponding 95% confidence interval for $\hat{\delta}_{\text{rem}}$ is given by $[-u_{\Delta/\mu}, -\ell_{\Delta/\mu}]$. The interval $C_{\Delta/\mu}^{0.95}$ is indeed a 95% CI for Δ/μ since

$$\begin{aligned}\mathbb{P}[\tfrac{\ell_\Delta}{u_\mu} \leqslant -\delta_{\text{rem}} \leqslant \tfrac{u_\Delta}{\ell_\mu}] &= \mathbb{P}[\tfrac{\ell_\Delta}{u_\mu} \leqslant \tfrac{\Delta}{\mu} \leqslant \tfrac{u_\Delta}{\ell_\mu}] \\ &\geqslant \mathbb{P}[\{\ell_\Delta \leqslant \Delta \leqslant u_\Delta\} \cap \{\ell_\mu \leqslant \mu \leqslant u_\mu\}] \\ &= 1 - \mathbb{P}[\{\Delta \notin [\ell_\Delta, u_\Delta]\} \cup \{\mu \notin [\ell_\mu, u_\mu]\}] \\ &\geqslant 1 - (\mathbb{P}[\{\Delta \notin [\ell_\Delta, u_\Delta]\}] + \mathbb{P}[\{\mu \notin [\ell_\mu, u_\mu]\}]) \\ &\geqslant 0.95.\end{aligned}$$

Since the decomposition of the variance of the transformed variable $\varphi(Z)$ into $\mathrm{Var}(\varphi(Z)) = \sigma_{\text{rem}}^2 + \sigma^2$ does not allow for an additive decomposition of the variance of Z, we report estimates and 95% CIs for $\sigma_{\text{rem}}/\hat{\mu}$ and $\sigma/\hat{\mu}$. We choose to normalise by the mean $\hat{\mu}$ of the transformed response variable before cell removal in order to get comparable results across the different response variables.

REFERENCES

Oehlert GW. *A first course in design and analysis of experiments* (Retrieved from the University of Minnesota Digital Conservancy) (2010).

Limbert E, Stahel W, Abbt M. Log-normal distributions across the sciences: Keys and clues. *BioScience* **51** (2001) 341–. doi:10.1641/0006-3568(2001)051[0341:LNDATS]2.0.CO;2.

Hosseini R. Quantiles equivariance (2010).
